# Influence of temporomandibular disorder on temporal and masseter muscles and occlusal contacts in adolescents: an electromyographic study

**DOI:** 10.1186/1471-2474-15-123

**Published:** 2014-04-10

**Authors:** Leandro Lauriti, Lara Jansiski Motta, Camila Haddad Leal de Godoy, Daniela Aparecida Biasotto-Gonzalez, Fabiano Politti, Raquel Agnelli Mesquita-Ferrari, Kristianne Porta Santos Fernandes, Sandra Kalil Bussadori

**Affiliations:** 1 Oral Surgery, University Nove de Julho (UNINOVE), São Paulo, SP, Brazil; 2 Paediatric Dentistry, University Nove de Julho (UNINOVE), São Paulo, SP, Brazil; 3 Rehabilitation Sciences, University Nove de Julho (UNINOVE), Av. Divino Salvador, 638 - Moema, CEP: 04078-012 São Paulo, SP, Brazil; 4 Master’s Course in Rehabilitation Sciences, University Nove de Julho (UNINOVE), São Paulo, SP, Brazil

**Keywords:** Temporomandibular joint, Occlusion, Occlusal contacts, Electromyography

## Abstract

**Background:**

The aim of the present study was to analyse the influence of temporomandibular disorder (TMD) on electromyographic activity in the masseter and temporal muscles of adolescents and investigate a possible association with the number of occlusal contacts.

**Methods:**

The Helkimo Index was administered for the diagnosis of TMD and classification of the adolescents into three groups: without TMD; with mild TMD; and with moderate/severe TMD. Carbon paper was used for the determination of occlusal contact points. A standardised electromyographic evaluation was performed on the masticatory muscles at rest, during habitual chewing and during maximum voluntary clenching. The readings were normalised to maximum voluntary clenching. Statistical analysis involved the chi-squared test and Fisher’s exact test. The Kruskal-Wallis test and one-way analysis of variance with Dunn’s post hoc test were used to compare differences between groups. Pearson’s correlation coefficients (*r*) were calculated for the determination of correlations between the number of occlusal contacts and RMS values.

**Results:**

Electromyography revealed significant differences in the right and left masseter and temporal muscles at rest and during chewing among the three groups. These differences were not observed during maximum voluntary clenching. No statistically significant differences were found between the groups with and without TMD regarding the number of occlusal contacts.

**Conclusion:**

Electromyographic activity in the masseter and temporal muscles was greater among adolescents with moderate to severe TMD.

## Background

Temporomandibular disorder (TMD) is characterised by clinical signs and symptoms that affect the masticatory muscles and temporomandibular joint [1]. Poor posture, malocclusion and bruxism (clenching/grinding of one’s teeth) can affect the masticatory muscles, temporomandibular joint and associated structures, giving rise to TMD [1,2]. Signs and symptoms of TMD include sensitivity in the muscles of the head and neck, pain in one or both temporomandibular joints, limited movement of the mandible, joint sounds, facial deformities and headache [2-6].

According to Okeson [7], occlusion is defined as the relationship between the upper and lower teeth in functional contact during activity of the mandible. Studies have suggested that determinants of functional changes in the stomatognathic system may lead to an imbalance among the occlusion, masticatory muscles and temporomandibular joint [8,9]. Moreover, TMD may be caused by occlusal macro-traumas and micro-traumas [10-14]. These conditions can cause mandibular deviations that affect chewing function and result in excessive pressure on the joint [15-18].

TMD is more prevalent among adults and may be related to high degrees of stress during activities of daily living as well as the prolonged maintenance of occlusal problems and parafunctional habits [3,6]. However, while the early diagnosis of TMD in young individuals is more difficult due to the lesser intensity of signs and symptoms, a growing number of studies have reported the occurrence of this disorder in the younger population [11,14]. Thus, the study of TMD in children and adolescents is essential to the early determination of problems that may predispose individuals to craniofacial growth abnormalities, pain and muscle dysfunction in adulthood [2-6].

The number of occlusal contact points in the paediatric population with and without TMD has been investigated as a way of diagnosing this disorder. A recent study has demonstrated that the number of occlusal contacts among children and adolescents with some degree of TMD is greater than among those without TMD [19].

Electromyography (EMG) is a complementary tool that can contribute knowledge on muscle physiology and assist in the differential diagnosis and monitoring of TMD [20,21]. In some studies, an increase in muscle activity has also been associated with an increase in the number of occlusal contacts [22,23].

The aim of the present study was to analyse the influence of TMD on the electromyographic activity of the masseter and temporal muscles in adolescents and investigate a possible association between the activity of these muscles and number of occlusal contacts.

## Methods

### Subjects

A convenience sample of adolescents attending the José de Paiva Netto Educational Institute in the city of Sao Paulo (Brazil) was evaluated. The sample size was calculated based on a pilot study using the DIMAM 1.0 sample calculation program and the root mean square (RMS) of the electromyographic signals of the masseter muscles and anterior bundles of the temporal muscles. Considering α = 0.05, β = 0.20 and an 80% test power, a minimum of 11 participants in each group was determined. To maintain a margin of safety, 14 individuals were selected for each group (without TMD, with mild TMD and with moderate to severe TMD). The following were the inclusion criteria: statement of informed consent signed by parent/guardian, presence of four first and second permanent molars, no deciduous teeth in the oral cavity and age between 14 and 18 years. The exclusion criteria were current or past orthodontic treatment, current medical or psychological treatment, periodontal problems and cavitated and/or extracted teeth. This study was conducted in compliance with the principles of the Declaration of Helsinki and received approval from the Human Research Ethics Committee of the University Nove de Julho (Brazil) under protocol number 332780.

The Helkimo questionnaire was administered for the evaluation of TMD. While the Research Diagnostic Criteria for Temporomandibular Disorders is more indicated for this classification, the Helkimo questionnaire is more easily understood by the paediatric population. Moreover, the Helkimo index has frequently been employed for the diagnosis of TMD [10,24-26]. On a specific chart, data were recorded from a clinical exam involving the extra-oral and intra-oral inspection of the teeth, type of occlusion and occlusal abnormalities as well as palpation of the masticatory muscles and temporomandibular joint. The analysis of mandibular movements involved the use of a digital calliper for the measurement of maximum mouth opening, lateral movements and protrusion. Joint sounds, headache, facial pain, difficulty chewing, breathing pattern and parafunctional habits were also investigated. Based on the criteria of the Helkimo Index, the participants were classified into the following groups: without TMD (WG); with mild TMD (MG); with moderate to severe TMD (MSG).

### Occlusal contacts

Carbon paper (Bausch® BK 01) was used for the determination of occlusal contact points on both sides simultaneously. For such, the participant was positioned with Camper’s plane parallel to the ground and instructed to bite down until achieving maximum clenching. The contact points were recorded on an occlusogram.

### Surface EMG recording

Surface EMG signals were recorded in the right and left masseter and anterior temporal bundles with disposable surface electrodes (Ag/AgCl - Medical Trace®) measuring 10 mm in diameter attached over the belly of the muscle in the region of the greatest tonus (determined during maximum voluntary clenching). Bandage tape was used to secure the electrodes further, with care taken to avoid micro-movements. The inter-electrode distance was 20 mm from centre to centre [27]. Prior to attachment, the sites for the electrodes were cleaned with a cotton ball soaked in alcohol to diminish impedance [28]. A reference electrode (3 x 2 cm) coated with Lectron II conductive gel (Pharmaceutical Innovations®) was attached to the left wrist of the volunteer to allow the recording of differential signals.

The bipolar EMG signals were amplified using an eight-channel module (EMG System do Brasil Ltda®), with a band pass filter with cut-off frequencies at 20–1000 Hz, an amplifier gain of 1000 fold and a common mode rejection ratio > 120 dB. The EMG signal was sampled through a 16- bit analogue-digital converter with a sampling rate of 2 kHz.

### Experimental protocol

The participants were instructed to remain seated in a chair, feet apart, shoulders relaxed, hands resting on thighs and with the head positioned such that the Frankfurt plane was parallel to the ground. The participants received no visual feedback of the signals registered on the computer.

To standardise the EMG potentials of the four muscles analysed with occlusal contacts, two strips of Parafilm M® (American National Can TM, Chicago, USA) folded into five parts (10 mm in thickness) were positioned on the mandibular first and second molars (bilaterally) of each subject [29]. Maximum voluntary clenching (MVC) was recorded for four seconds three times with a three-minute interval between readings.

After five minutes of rest, EMG activity was recorded for 15 seconds three times successively each under the following conditions: i) *Resting position*: the subject was asked to relax and maintain the maxillary and mandibular teeth out of contact; ii) *maximum voluntary clenching* (isometric): the subject was instructed to clench as hard as possible and maintain this level of contraction; and iii) *chewing* (isotonic): the subject was instructed to lightly and systematically bite down on the two strips of parafilm positioned bilaterally in time with a metronome calibrated to 60 beats per minute. A three-minute interval between readings was used for the EMG signals recorded in the resting position and during chewing and a five-minute interval between readings was used for MVC.

### EMG data analysis

Ten seconds of the signal were used for the calculation of the root mean square (RMS) amplitude, with the initial three seconds and final two seconds of the 15-second reading discarded. For data recorded in the resting position and during MVC, the RMS was calculated using a 200-ms moving window. The amplitude of the signal under all three conditions was expressed as the percentage of the maximum RMS potential recorded in the three readings of MVC (%MVC). All EMG signal processing was performed using specific routines carried out on the Matlab program, version 7.1 (MathWorks Inc., Natick, Massachusetts, USA).

### Statistical analysis

Statistical analysis involved the chi-squared test and Fisher’s exact test for the categorical variables. RMS data were expressed as mean and standard deviation (SD) values. Same-day reproducibility of the EMG values for each muscle and group (WG, MG and MSG) was estimated through test-retest reliability analyses, considering three tests per subject. Intra-class correlation coefficients (ICC) greater than 0.8 indicate excellent reproducibility, values between 0.6 and 0.8 denote good reproducibility and values below 0.6 reflect poor reproducibility [30]. Depending on the distribution of the data (normal or non-normal), either one-way analysis of variance with Dunn’s post hoc test or the nonparametric Kruskal-Wallis test were used to examine differences among groups (WG, MG and MSG). Spearman’s correlation coefficients (*r*) were calculated for the determination of correlations between the number of occlusal contacts and RMS values. The Mann–Whitney *U* test was used to compare pain between the MG and MSG based on the Helkimo questionnaire. The level of significance of each comparison was set to 5% (*p* < 0.05). All statistical analyses were conducted using the SPSS program, version 12.0 (SPSS Inc., Chicago, USA).

## Results

### Distribution of volunteers: TMD and gender

Eighty-one adolescents aged 14 to 18 years were evaluated [mean age: 15.64 years (SD: 1.06)]; 51.9% (n = 42) were males and 48.1% (n = 39) were females. The prevalence of TMD was 74.1% (n = 60). The prevalence of TMD among females was 87.2% (n = 34). A statistically significant association was found between the female gender and TMD (Pearson’s chi-squared test = 6.727; p = 0.009). Mild TMD predominated among males (54.8%; n = 23). Among the females, mild TMD was found in 46.2% (n = 18), moderate TMD was found in 35.9% (n = 14) and severe TMD was found in 5.1% (n = 2). A statistically significant association was also found between the female gender and severe TMD (Fisher’s exact test = 15.399, p = 0.002) (Table 1).

**Table 1:** Distribution of volunteers according to degree of TMD and gender

			**TMD**				**Total**
			**Absent**	**Mild**	**Moderate**	**Severe**	
Gender	Male	N	16	23	3	0	42
		%	38.1%	54.8%	7.1%	0.0%	100.0%
	Female	N	5	18	14	2	39
		%	12.8%	46.2%	35.9%	5.1%	100.0%
Total		N	21	41	17	2	81
		%	25.9%	50.6%	21.0%	2.5%	100.0%

Fisher’s exact test.

### Pain

According to Helkimo [31], muscle pain and pain in the temporomandibular joint are frequent symptoms of TMD. Thus, the influence of pain in the MG and MSG was compared based on two questions of the Helkimo index: Q1: Do you have pain in the TMJ or in the area of masticatory muscles? Q2: Do you have locked mandible during opening the mouth? The comparisons demonstrated significantly greater pain in the MSG (mean ± SD: Q1 = 9.66 ± 3.70; Q2 = 8.66 ± 2.86) in comparison to the MG (mean ± SD: Q1 = 4.00 ± 4.00; Q2 = 3.15 ± 3.70) for both questions (Q1: *p* < 0.003; *U*-test = 40.00, Q2: *p* < 0.003; *U*-test = 50.00).

### Intra-session reliability of EMG data

Table 2 displays the estimated ICCs for the EMG values of each muscle under each condition considering three tests per subject. Good to excellent reproducibility was demonstrated (ICC: 0.66 to 0.99).

**Table 2:** Intra-class correlation coefficient calculated for subjects without TMD (WG), with mild TMD (MG) and with moderate to severe TMD (MSG)

	**Resting position**			**Maximum voluntary clenching**			**Chewing**		
	**WG**	**MG**	**MSG**	**WG**	**MG**	**MSG**	**WG**	**MG**	**MSG**
**Right temporal**	0.88	0.92	0.95	0.74	0.87	0.66	0.95	0.88	0.78
**Right masseter**	0.80	0.70	0.99	0.80	0.72	0.71	0.87	0.71	0.69
**Left temporal**	0.95	0.92	0.96	0.87	0.74	0.83	0.74	0.82	0.75
**Left masseter**	0.76	0.83	0.98	0.86	0.75	0.67	0.85	0.82	0.91

### EMG activity

**Figure 1:**
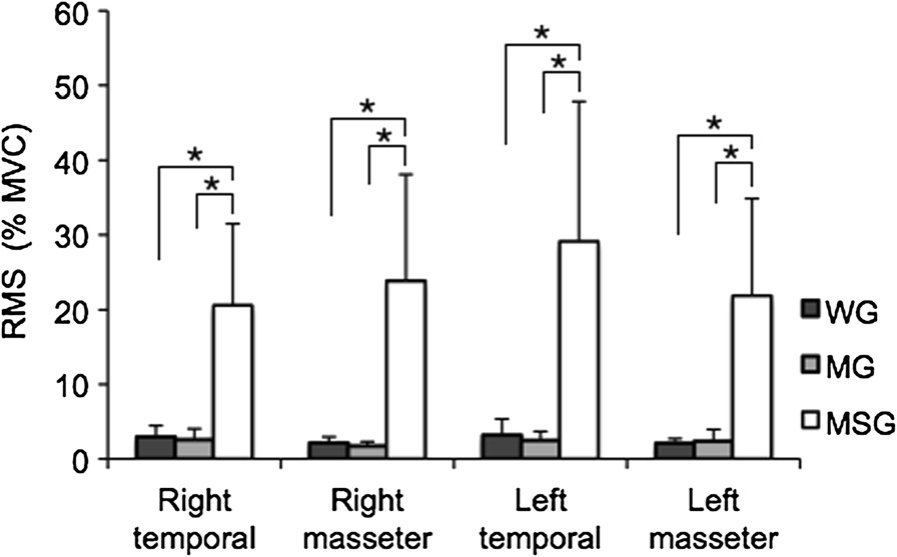
Mean and SD of RMS (%MVC) value recorded in resting position; RMS of right and left masseter and termporal muscles compared between without TMD (WG), with mild TMD (MG) and with moderate to severe TMD (MSG); *Significant difference (*p * **< 0.05; Dunn’s post-hoc test).**

**Figure 2:**
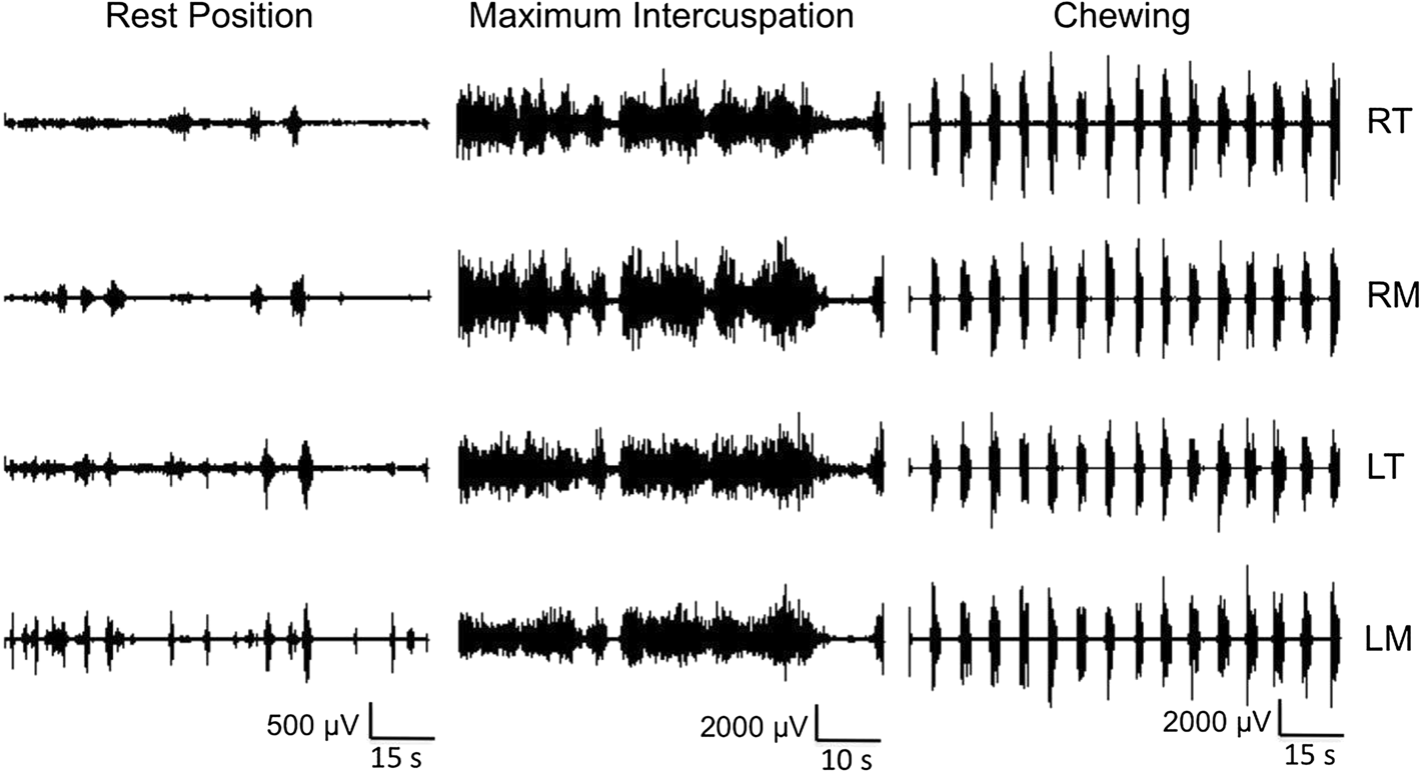
Typical raw EMG signal of right temporal (RT) and masseter (RM) muscles and left temporal (LT) and masseter (LM) muscles from patient in MSG recorded in resting position, maximum intercuspation and chewing.

Figure 1 displays a typical electromyogram from the MSG. Significant differences in RMS values in the right and left masseter and temporal muscles were found among the three groups in the resting position (Table 3), whereas no differences were found during MVC or chewing. In the resting position (Figure 2), RMS values in the right and left masseter and temporal muscles were significantly higher in the MSG in comparison to the other two groups, whereas no significant differences were found between the WG and MG.

**Table 3:** Mean (SD) of the RMS values (%MVC) of right and left masseter and temporal muscles in individuals without TMD (WG), with mild TMD (MG) and with moderate to severe TMD (MSG)

	**WG**	**MG**	**MSG**	***p-*value**
***Resting position***				
Right temporal	2.93 ± 1.53	2.55 ± 1.49	20.61 ± 10.87	<0.001*
Right masseter	2.14 ± 0.81	1.71 ± 0.59	23.85 ± 14.25	<0.001*
Left temporal	3.18 ± 2.17	2.47 ± 1.21	29.11 ± 18.78	<0.001*
Left masseter	2.08 ± 0.63	2.35 ± 1.59	21.89 ± 12.97	<0.001*
***Maximum voluntary clenching***				
Right temporal	74.17 ± 8.60	72.20 ± 20.12	68.95 ± 8.37	0.10
Right masseter	67.59 ± 27.41	66.32 ± 66.83	66.83 ± 11.12	0.77
Left temporal	70.49 ± 11.61	75.81 ± 11.50	67.18 ± 12.75	0.21
Left masseter	74.07 ± 12.86	68.40 ± 11.02	64.99 ± 7.94	0.44
***Chewing***				
Right temporal	36.83 ± 13.95	42.31 ± 17.29	45.48 ± 13.03	0.32
Right masseter	38.02 ± 15.13	44.44 ± 17.34	46.11 ± 14.15	0.40
Left temporal	38.02 ± 15.13	44.44 ± 17.34	46.11 ± 14.15	0.51
Left masseter	35.03 ± 12.11	44.76 ± 15.94	46.62 ± 18.82	0.15

*Significant difference (*p* < 0.05; Kruskal-Wallis test).

### EMG activity and occlusal contacts

RMS values of the right and left temporal and masseter muscles were not correlated with the ipsilateral number of occlusal contacts (right or left side) (Table 4).

**Table 4:** **Spearman’s correlation coefficients (*r*) for occlusal contacts (OC) and RMS (% MVC) in individuals without TMD (WG), with mild TMD (MG) and with moderate to severe TMD (MSG)**

	**WG**		**MG**		**MSG**	
	***r***	***p*-value**	***r***	***p*-value**	***r***	***p*-value**
Right temporal	0.03	0.89	0.18	0.51	−0.10	0.71
Right masseter	0.22	0.44	0.30	0.29	−0.44	0.11
Left temporal	0.45	0.74	0.28	0.31	0.17	0.55
Left masseter	0.48	0.20	0.20	0.47	0.22	0.43

## Discussion

In the present study, the prevalence of TMD in the female gender was 87.2%. The literature reports that the greater frequency of this disorder among females is due to hormonal, postural, emotional, occlusal and functional factors as well as genetic predisposition [11,13,14].

Regarding the EMG activity of the right and left masseter and temporal muscles, a significantly higher of the RMS amplitude in the rest position was found in the MSG in comparison to the WG and MG, whereas no significant differences among groups were found regarding MVC or chewing. While pain in the present study was determined only through the Helkimo index, the results found in the resting position may have been influenced by the greater degree of muscle pain and pain in the temporomandibular joint in the MSG. Similar results (greater EMG activity in the right and left masseter and temporal muscles of patients with pain in comparison to a control group) have been described in a previous study [8].

In general, emotional aspects such as anxiety, anger and frustration, may trigger oral habits, such as bruxism (clenching/grinding the teeth), leading to an increase in the activity of the masticatory muscles (especially the masseter and temporal muscles), with consequent hypertonia, muscle pain and an aggravation of TMD [32,33]. As these symptoms are more evident in individuals with more severe degrees of TMD, the discontinuous bursts of EMG activity in the right and left masseter and temporal muscles in the resting position in the MSG (Figure 1) may be the consequence of hypertonia in these muscles. However, no firm conclusion can be drawn regarding this issue, as no previous studies have reported similar EMG signals to those found in the present investigation for individuals with moderate to severe TMD.

The determination of EMG activity in the temporal and masseter muscles with the mandible at rest is of fundamental importance to comparisons between individuals, as muscle activity in the resting position is dependent on the lengthening reflex and is effectively maintained by the tonicity of the muscles that counterbalance the action of gravity and negative intra-oral pressure [34]. However, the physiological basis of the mandibular resting position is one of the most controversial areas in oral physiology [35,36]. In the resting position, the muscles are slightly contracted and EMG activity is greater than in the physiological resting position. In a recent study, standardised EMG activity was determined in the masseter and temporal muscles in women with and without TMD and the authors found that those with TMD exhibited greater asymmetry between muscle pairs as well as hyperactivity of the masseter muscle [37].

In a study carried out by Ferrario et al. [38] involving 27 healthy men and 35 healthy women divided into two groups (one with bilateral, symmetrical [complete] Angle class I with a class I relationship between canines and molars and another with partial Angle class I as well as classes II and III), the authors concluded that complete or partial Angle class I seems not to affect the electromyographic activity of the masseter, temporal or sternocleidomastoid muscles during MVC. Individuals with complete Angle class I had a tendency to be more homogeneous than individuals with partial Angle class I [38]. Due to the methodological limitations of the study, evaluations of larger groups are needed to draw more consistent conclusions. In the present study, no correlation was found between the number of ipsilateral occlusal contacts and EMG signals in the masseter or temporal muscles during MVC or chewing.

In an EMG analysis of the masseter and anterior temporal muscles in 40 male and female youths and adults without TMD, variations were found in the amplitude of the signal in accordance with the composition, type and size of the muscle fibres during MVC [39]. The study cited found significant differences between males and females in mean power frequency and the authors state that EMG is an important tool for the study of adaptive functional alterations. In the present investigation, greater EMG activity was found in the masseter and temporal muscles in the group with moderate to severe TMD, with significant differences in relation to the other groups at rest, but not during habitual chewing or MVC.

In one study, 30 young patients with long-standing TMD and 20 young healthy individuals were evaluated through standardised EMG of the masseter and temporal muscles during MVC. The patients with TMD exhibited increased and more asymmetric activity in the anterior temporal muscle and reduced mean power frequencies in comparison to the controls. Mean frequencies in the temporal muscles were greater than those in the masseter muscles in both groups [40].

Another study performed qualitative and quantitative comparisons of normalised and non-normalised (absolute data) EMG amplitude values in the masseter and anterior temporal muscles during habitual chewing among 41 women (21 asymptomatic and 20 symptomatic for TMD) to determine whether normalisation would affect the interpretation of the clinical findings. Activity in the masseter muscle was significantly lower in the group with TMD. After the normalisation of the data, however, no significant differences between groups were found. These results suggest that absolute data may represent clinical findings in the qualitative analysis of the signal [41]. Another study involving dental students found a considerable reduction in EMG activity in the masticatory muscles with the mandible at rest and positioned at a few millimetres of inter-occlusal distance [42].

One study compared the electrical signals of the masseter and temporal muscles at rest as well as under isotonic and isometric conditions and analysed TMJ sounds during opening and closing of the mouth in a group with TMD before and after treatment. The authors found a reduction in vibratory intensity after treatment, whereas the electrical activity increased in the masseter muscle and decreased in the temporal muscle under isotonic and isometric conditions. However, the differences did not achieve statistical significance [43]. EMG studies demonstrate that TMD causes an alteration in muscle activity, with a reduction in strength, especially on the side not used for chewing [44]. This reduction in strength has been attributed to anxiety and muscle shortening.

Premature occlusal contacts are a possible cause of headaches, facial pain and TMD, affecting chewing function and causing asymmetry in the stomatognathic system [45-47]. This can have secondary consequences stemming from the change in occlusal position due to joint and muscle pain, causing deviation of the mandible and leading to excessive pressure on the joint and bilaminar zone [47]. In the growth phase, occlusal interference can lead to symptoms due to the functional deviation of the mandible and periodontal alterations stemming from pain. This functional alteration between dental arches can exert a negative neuromuscular influence, with severe craniocervical disorders that can lead to the development of TMD and directly affect the severity of the condition, with a consequent impact on quality of life [37,40,46-49].

Muscle conditions are progressive and have a variety of aetiological factors, such as occlusal interference and isometric contractions, with the retention of fluids in the muscle tissue, a reduction in blood supply and the build-up of metabolic products. It is known that an improvement in the condition of the masticatory muscles leads to enhanced chewing capacity [50]. The results of the present study reveal that physiological muscle alterations are present in adolescents with TMD, but no significant correlation was found with the number of occlusal contacts.

The present study has limitations that should be addressed. Although the use of carbon paper for the record of occlusal contacts is the gold standard in the literature, this method may not be accurate when used on young volunteers. Future studies should record these contact points with the use of an occlusal sensor. Another limitation was the failure to include parafunctional habits among the exclusion criteria. Due to the statistical limitations of the study, evaluations of larger groups are needed to draw firmer conclusions.

## Conclusion

Statistically significant differences in electromyographic activity of the masseter and temporal muscles were found in the resting position in adolescents with moderate to severe TMD in comparison to those with mild TMD and those without TMD. However, no statistically significant differences were found between groups with and without TMD regarding the number of occlusal contacts.

## Competing interests

All authors declare that they have no competing interests.

## Authors’ contributions

LL conceived and designed the study, participated in the data acquisition, performed the analysis and interpretation of the data and drafted the manuscript. SKB performed a critical review of the manuscript for intellectual content. CHLG, LJM, RAMF and KPSF participated in the data acquisition and drafting of the manuscript. DABG and FP performed the analysis and interpretation of data and performed a critical review of the manuscript for intellectual content. All authors read and approved the final manuscript.

## Pre-publication history

The pre-publication history for this paper can be accessed here:

http://www.biomedcentral.com/1471-2474/15/123/prepub

## References

[B1] Catanzarati JF Debuse T Duquesnoy B Chronic neck pain and masticatory dysfunction Joint Bone Spine 2005 72 6 515 519 10.1016/j.jbspin.2004.10.007 16226475

[B2] Thilander B Rubio G Pena L Mayorga C Prevalence of temporomandibular disordens and its association with malocclusion in children and adolescents: an epidemiologic study related to specified stage of dental development Angle Orthod 2002 72 2 146 154 11999938 10.1043/0003-3219(2002)072<0146:POTDAI>2.0.CO;2

[B3] Manfredini D Castroflorio T Perinetti G Guarda- Nardini L Dental occlusion, body posture and temporomandibular disorders: where we are now and where we are heading for J Oral Rehabil 2012 39 6 463 471 10.1111/j.1365-2842.2012.02291.x 22435603

[B4] Solow B Sonnesen L Head posture and malocclusions Eur J Orthod 1998 20 3 685 693 9926635 10.1093/ejo/20.6.685

[B5] Sonnesen L Bakke M Solow B Temporomandibular disorders in relation to craniofacial dimensions, head posture and bite force in children selected for orthodontic treatment Eur J Orthod 2001 23 2 179 192 10.1093/ejo/23.2.179 11398555

[B6] Tsai CM Chou SL Gale EN McCall WD Jr Human masticatory muscle activity and jaw position under experimental stress J Oral Rehabil 2002 29 1 44 51 10.1046/j.1365-2842.2002.00810.x 11844031

[B7] Okeson JP Orofacial pain guidelines assessment, diagnosis and managements 1996 Chicago: Ed. Quintessence

[B8] Liu ZJ Yamagata K Kasahara Y Ito G Electromyographic examination of jaw muscles in relation to symptoms and occlusion of patients with temporomandibular joint disorders J Oral Rehabil 1999 26 1 33 47 10.1046/j.1365-2842.1999.00356.x 10080323

[B9] Nassif NJ Al-Salleeh F Al-Admawi M The prevalence and treatment needs of symptoms and signs of temporomandibular disorders among young adult males J Oral Rehabil 2003 30 9 944 950 10.1046/j.1365-2842.2003.01143.x 12950977

[B10] Bourzgui F Sebbar M Nadour A Hamza M Prevalence of temporomandibular dysfunction in orthodontic treatment Int Orthod 2010 8 4 386 398 10.1016/j.ortho.2010.09.003 21093399

[B11] Ciancaglini R Gherlone EF Redaelli S Redaelli G The distribuition of oclusal contacts in the intercuspal position and temporomandibular disorder J Oral Rehabil 2002 29 11 1082 1090 10.1046/j.1365-2842.2002.00941.x 12453263

[B12] He SS Deng X Wamalwa P Chen S Correlation between centric relation and maximum intercuspation discrepancy and temporomandibular joint dysfunction Acta Odontol Scand 2010 68 6 368 376 10.3109/00016357.2010.517552 20942605

[B13] Kafas P Leeson R Assessment of pain in temporomandibular disorders: the bio-psychosocial complexity Int J Oral Maxillofac Surg 2006 35 2 145 149 10.1016/j.ijom.2005.04.023 15975765

[B14] Magnusson T Egermark I Carlsson GE A longitudinal epidemiologic study of signs and symptoms of temporomandibular disorders from 15 to 35 years of age J Orofac Pain 2000 14 4 310 319 11203765

[B15] Bonakdarchian M Askari N Askari M Effect of face form on maximal molar bite force with natural dentition Arch Oral Biol 2009 54 3 201 204 10.1016/j.archoralbio.2008.11.009 19131047

[B16] Fujii T Occlusal conditions just after the relief of temporomandibular joint and masticatory muscle pain J Oral Rehabil 2002 29 4 323 329 10.1046/j.1365-2842.2002.00835.x 11966964

[B17] Hatch JP Shinkai RS Sakai S Rugh JD Paunovich ED Determinants of masticatory performance in dentate adults Arch Oral Biol 2001 46 7 641 648 10.1016/S0003-9969(01)00023-1 11369319

[B18] Miyawaki S Tanimoto Y Araki Y Katayama A Kuboki T Takano-Yamamoto T Movement of the lateral and medial poles of the working condyle during mastication in patients with unilateral posterior crossbite Am J Orthod Dentofacial Orthop 2004 126 5 549 554 10.1016/j.ajodo.2003.10.033 15520687

[B19] Santis TO Motta LJ Guedes CC Santos Z Jr Fernandes KPS Mesquita-Ferrari RA Bussadori SK Study of occlusal contact in children with temporomandibular disorder- pilot study Eur J Paediatr Dent 2012 13 2 97 100 22762169

[B20] Ferrario VF Serrao G Dellavia C Caruso E Sforza C Relationship between the number of occlusal contacts and masticatory muscle activity in healthy young adults Cranio 2002 20 2 91 98 12002835 10.1080/08869634.2002.11746196

[B21] Serrao G Sforza C Dellavia C Antinori M Ferrario VF Relation between vertical facial morphology and jaw muscle activity in healthy young men Prog Orthod 2003 4 45 51 10.1034/j.1600-9975.2002.02031.x 12887579

[B22] Amorim CF Giannasi LC Ferreira LM Magini M Oliveira CS de Oliveira LV Hirata T Politti F Behavior analysis of electromyographic activity of the masseter muscle in sleep bruxers J Bodyw Mov Ther 2010 14 3 234 238 10.1016/j.jbmt.2008.12.002 20538220

[B23] Watanabe K The relationship between dentofacial morphology and the isometric jaw-opening and closing muscle function as evaluated by electromyography J Oral Rehabil 2000 27 7 639 645 10.1046/j.1365-2842.2000.00541.x 10931258

[B24] Ardizone I Celemin A Aneiros F del Rio J Sanchez T Moreno I Electromyographic study of activity of the masseter and anterior temporalis muscles in patients with temporomandibular joint (TMJ) dysfuction: comparison with the clinical dysfunction index Med Oral Patol Oral Cir Bucal 2010 15 1 e14 e19 19767710 10.4317/medoral.15.e14

[B25] Leuin SC Frydendall E Gao D Chan KH Temporomandibular joint dysfunction after mandibular fracture in children: a 10-year review Arch Otolaryngol Head Neck Surg 2011 137 1 10 14 10.1001/archoto.2010.237 21242539

[B26] Perillo L Femminella B Farronato D Baccetti T Contardo L Perinetti G Do malocclusion and Helkimo Index ≥ 5 correlate with body posture? J Oral Rehabil 2011 38 4 242 252 10.1111/j.1365-2842.2010.02156.x 21070327

[B27] Hermens HJ Freriks B Merletti R Stegeman D Blok J Rau G Disselhorst-Klug C Hägg G European recommendations for surface eletromyography – SENIAM. Book 8 1999 Enschede (Netherlands): Roessingh Research and Development

[B28] De Luca CJ The use of surface electromyography in biomechanics J App Biomech 1997 13 2 135 163

[B29] Biasotto-Gonzalez DA Berzin F da Costa JM de Gonzalez TO Electromyographic study of stomatognathic system muscles during chewing of different materials Electromyogr Clin Neurophysiol 2010 50 2 121 127 20405788

[B30] Bartko JJ The intraclass correlation coefficient as a measure of reliability Psychol Rep 1966 19 3 11 10.2466/pr0.1966.19.1.3 5942109

[B31] Helkimo M Studies of function and dysfunction of the masticatory system II Index for anamnestic and clinical dysfunction and occlusal state Swed Dent J 1974 67 101 121 4524733

[B32] Rugh JD Solberg WK Psychological implications in temporomandibular pain and dysfunction Oral Sci Rev 1976 7 3 30 775369

[B33] Gomes EA Garcia AR Zuim PRJ Costa PS Mandibular rest position: a literature review Rev Odontol Araçatuba 2006 27 2 81 86

[B34] Sgobbi De Faria CRS Berzin F Electromyographic study of the temporal, masseter and suprahyoid muscles in the mandibular rest position J Oral Rehabil 1998 25 10 776 780 10.1046/j.1365-2842.1998.00312.x 9802586

[B35] Miles TS Postural control of the human mandible Arch Oral Biol 2007 52 4 347 352 10.1016/j.archoralbio.2006.12.017 17257577

[B36] Van der Glas HW van der Bilt A Abbink JH Mason AG Cadden SW Functional role of oral reflexes in chewing and biting: phase-, task- and site-dependent reflex sensitivity Arch Oral Biol 2007 52 4 365 369 10.1016/j.archoralbio.2006.10.022 17129573

[B37] Wessberg GA Epker BN Elliott AC Comparison of mandibular rest positions induced by phonetics, transcutaneous electrical stimulation, and masticatory electromyography J Prosthet Dent 1983 49 1 100 105 10.1016/0022-3913(83)90248-2 6571889

[B38] De Felício CM Ferreira CL Medeiros AP Rodrigues Da Silva MA Tartaglia GM Sforza C Electromyographic indices, orofacial myofunctional status and temporomandibular disorders severity: a correlation study J Electromyogr Kinesiol 2012 22 2 266 272 10.1016/j.jelekin.2011.11.013 22206640

[B39] Ferrario VF Tartaglia GM Galletta A Grassi GP Sforza C The influence of occlusion on jaw and neck muscle activity: a surface EMG study in healthy young adults J Oral Rehabil 2006 33 5 341 348 10.1111/j.1365-2842.2005.01558.x 16629892

[B40] Gadotti IC Bérzin F Biasotto-Gonzalez DA Preliminary rapport on head posture and muscle activity in subjects with class I and II J Oral Rehabil 2005 32 11 794 799 10.1111/j.1365-2842.2005.01508.x 16202042

[B41] Tartaglia GM Lodetti G Paiva G De Felicio CM Sforza C Surface electromyographic assessment of patients with long lasting temporomandibular joint disorder pain J Electromyogr Kinesiol 2011 21 4 659 664 10.1016/j.jelekin.2011.03.003 21463956

[B42] Kroll CD Bérzin F Alves MC Clinical evaluation of masticatory muscles activity during habitual mastication: a study about normalization of electromyographic data Rev Odontol UNESP 2010 39 3 157 162

[B43] Michelotti A Farella M Vollaro S Martina R Mandibular rest position and electrical activity of the masticatory muscles J Prosthet Dent 1997 78 1 48 53 10.1016/S0022-3913(97)70087-8 9237146

[B44] Turcio KHL Garcia AR Derogis AR Zuim PRJ Electromyographic and electrovibratographic evaluation before and after TMJ treatment Pós Grad Rev Odontol 2002 5 2 36 43

[B45] Tsolka P Fenlon MR McCullock AJ Preiskel HW A controlled clinical, eletromyographic, and kinesiographic assesment of craniomandibular disorders in women J Orofac Pain 1994 8 1 80 89 8032335

[B46] Feteih RM Signs and symptoms of temporomandibular disorders and oral parafunctions in urban Saudi Arabian adolescents: a research report Head Face Med 2006 2 25 10.1186/1746-160X-2-25 16914032 PMC1563458

[B47] Laimi K Vahlberg T Salminen J Metsähonkala L Mikkelsson M Anttila P Aromaa M Sillanpãã M Does neck pain determine the outcome of adolescent headache? Cephalalgia 2007 27 3 244 253 10.1111/j.1468-2982.2006.01266.x 17381557

[B48] Troeltzsch M Troeltzsch M Cronin RJ Brodine AH Frankenberger R Messlinger K Prevalence and association of headaches, temporomandibular joint disorders, and occlusal interferences J Prosthet Dent 2011 105 6 410 417 10.1016/S0022-3913(11)60084-X 21640243

[B49] Motta LJ Martins MD Fernandes KP Mesquita-Ferrari RA Biasotto-Gonzalez DA Bussadori SK Craniocervical posture and bruxism in children Physiother Res Int 2011 16 1 57 61 10.1002/pri.478 21110415

[B50] Kimoto K Fushima K Tamaki K Toyoda M Sato S Uchimura N Assymetry of masticatory muscle activity during the closing phase of mastication Cranio 2000 18 4 257 263 11202845 10.1080/08869634.2000.11746139

